# A retrospective evaluation of the relationship between symmetric dimethylarginine, creatinine and body weight in hyperthyroid cats

**DOI:** 10.1371/journal.pone.0227964

**Published:** 2020-01-28

**Authors:** Donald Szlosek, Jane Robertson, Jessica Quimby, Rebekah Mack, Jennifer Ogeer, Celeste Clements, Donald J. McCrann, Michael J. Coyne

**Affiliations:** 1 IDEXX Laboratories, Inc., Westbrook, Maine, United States of America; 2 Department of Veterinary Clinical Sciences, The Ohio State University, Columbus, Ohio, United States of America; Tokushima University Graduate School, JAPAN

## Abstract

Hyperthyroidism in cats can mask changes in renal function, including chronic kidney disease (CKD), because of hyperfiltration and muscle loss. Symmetric dimethylarginine (SDMA) has been shown to increase earlier than creatinine in cats with renal dysfunction, and, unlike creatinine, SDMA is not impacted by lean muscle mass. The aim of this study was to describe the relationship between SDMA, creatinine, body weight and TT4 over time during treatment of hyperthyroidism. Cats were retrospectively identified from the US IDEXX Reference Laboratories database where TT4, SDMA and creatinine were measured on the same cat at multiple time points. A hyperthyroid treated group was identified (TT4 ≤ 4.7 μg/dL and decreased by a minimum of 2.5 μg/dL) that had body weight and laboratory results available from more than one visit, and was used to evaluate body weight, creatinine, SDMA and TT4 pre-treatment and at 1–30, 31–60, 61–90, 91–120 days post-treatment. Creatinine significantly decreased with increasing concentrations of TT4 (Spearman’s ρ = -0.37, P < 0.001), whereas SDMA did not. Body weight, SDMA and creatinine concentrations significantly increased during the immediate 1–30 day post-treatment period (P < 0.012, P < 0.001, P < 0.001, respectively). During treatment creatinine continued to increase as cats gained weight. In contrast, SDMA remained stable during treatment and was comparable to age-matched control cats. Therefore, SDMA may be a more reliable biomarker of renal function than creatinine in hyperthyroid cats before and during treatment.

## Introduction

Hyperthyroidism is the most common endocrinopathy affecting the geriatric feline population, with a prevalence of 6 to 10 percent reported in cats greater than 10 years of age. [1,2]. Chronic kidney disease (CKD) is a common co-morbidity in this population; estimated to occur in 15 to 49 percent of hyperthyroid cats [3–8]. The hypermetabolic state caused by hyperthyroidism leads to increased glomerular filtration rate (GFR) and reduced muscle mass, often confounding the diagnosis of CKD [9,10]. These pathologic changes complicate finding a reliable and consistent marker for renal dysfunction in hyperthyroidism. The ability to identify cats with change in renal function prior to, or at the onset of treatment for, hyperthyroidism may influence the long-term management of hyperthyroidism, as well as influence renal health.

Creatinine (CREA) has been the most common renal biomarker used in the diagnosis and monitoring of renal dysfunction in cats. Loss of muscle mass results in decreased CREA [11]; this leads to difficulty interpreting CREA in hyperthyroid cats due to the degree of muscle wasting often present [9]. CREA can remain within the normal reference range in some cats with hyperthyroidism despite concurrent renal disease [12]. Symmetric dimethylarginine (SDMA) has been shown to be an earlier and more sensitive biomarker for the assessment of GFR and the evaluation of CKD in feline and canine patients [13–15]. SDMA is a byproduct of intranuclear arginine methylation, and is excreted primarily (greater than 90%) by renal clearance [16,17]. Because SDMA is excreted by the kidneys, serum concentrations are affected by changes in GFR [13,14,18]; however, unlike CREA, are not impacted by lean body mass [15,18]. Because SDMA is unaffected by lean body mass, it is hypothesized that SDMA would be a consistent indicator of kidney function in hyperthyroid cats undergoing treatment for hyperthyroidism.

The objective of this study was to use big data to describe the relationship between CREA and TT4, SDMA and TT4, and the changes observed in bodyweight, SDMA, and CREA during treatment of feline hyperthyroidism. A secondary objective of this study was to demonstrate the feasibility and utility of a data collection method referred to as big data. Big data uses statistical methods to take a large population with low information density and create interpretable data to better understand the relationships and dependencies within populations, and perform predictions of outcomes and behaviors. By aggregating large amounts of data and accounting for the multitude of inputs and layers a better picture of what constitutes a disease, trend, or result interpretation for an entire population can be developed [19]. In this study this methodology was used to collect a specific feline population for evaulation and outcome.

## Materials and methods

Data were obtained through the US IDEXX Reference Laboratories database from clinical case samples submitted from 13 July 2015 to 17 January 2017. Each study sample was obtained and submitted to an IDEXX commercial reference laboratory by a practicing veterinarian during the routine diagnostic workup or monitoring of clinically well and ill cats in his or her care. All samples were obtained on the consent of the pet owner. Each sample result was identified by the breed, age, and sex of the cat from which the sample was obtained. To ensure privacy, demographic information on the pet, pet owner, or veterinarian who submitted the sample was not collected.

**Fig 1:**
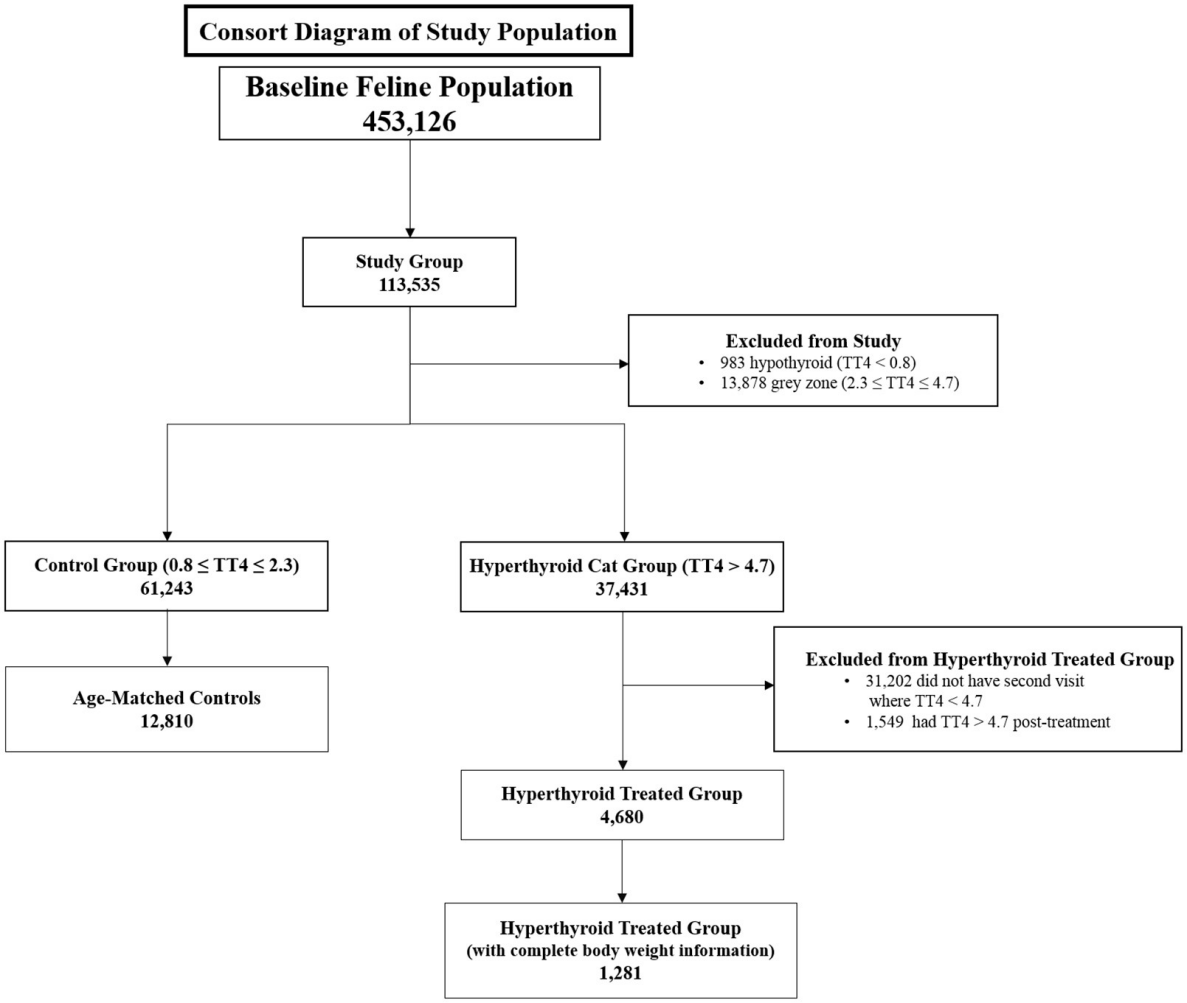
Consort diagram of study population.

Samples from a total of 453,126 individual cats were eligible for inclusion into the study. To be included in the study, cats needed a TT4, SDMA and CREA measured at least once on a single blood sample. For clarity, a consort diagram of the study population appears in Fig 1, and terms used in this study are defined in S1 Table.

A hyperthyroid cat group was identified from this sample population. For inclusion into the hyperthyroid cat group, cats had to be between 6 to 25 years of age and had to have at least a single blood sample with a TT4 concentration that was greater than 4.7 μg/dL (the upper reference interval for IDEXX Reference Laboratory) along with a SDMA and CREA concentration measured concurrently. Body weights (kg) were obtained when available for cats in the hyperthyroid cat group.

A hyperthyroid treated group was identified from the hyperthyroid cat group. For inclusion in the hyperthyroid treated group, hyperthyroid cats had to have samples from more than one visit in which the TT4, SDMA, and CREA were measured and demonstrated a change in TT4 concentrations in those samples using the following criteria: on the sample from the visit following their maximal TT4 concentration (> 4.7 μg/dL) the concentration of TT4 had to have decreased by a minimum of 2.5 μg/dL and be ≤ 4.7 μg/dL. Treatment was defined in this manner since individual therapy for each cat was unknown. The pre-treatment visit was defined as the first time at which the cat’s sample had a TT4 concentration > 4.7 μg/dL. The post-treatment visits were defined as all subsequent visits where the cat’s sample had a TT4 concentration ≤ 4.7 μg/dL with a minimum decrease of 2.5 μg/dL from pre-treatment levels. All cats had to have at least one post-treatment visit. For this study the post-treatment period was limited to 120 days. Cats with samples that remained hyperthyroid (TT4 > 4.7 μg/dL) during the post-treatment visits were excluded.

A control group was identified from the baseline feline population of 453,126 cats. For inclusion in the euthyroid control group, cats had to have samples for at least one visit in which the TT4, SDMA, and CREA were measured and have a TT4 concentration ≥ 0.8 μg/dL and < 2.3 μg/dL on all visits. Control cats with bodyweight information were selected at a 10:1 age-matched comparison with the hyperthyroid treated population that had complete body weight information.

The time (measured in days) from pre-treatment to the post-treatment visits was separated into 30-day increments to reflect clinical follow-up times. The binning of the post-treatment time periods was done *pre-hoc* and blinded to the results of the response variables. If a cat had more than one visit in a defined 30-day increment, the latest visit was used. These time periods (1–30 days, 31–60 days, 61–90 days, and 91–120 days) were used as timepoint comparisons for changes in BW, SDMA, and CREA. Pre-treatment population was compared to all time points, and each time point to each other to determine if any significant changes were present in BW, SDMA and CREA during treatment.

### Biomarkers

CREA, SDMA, and TT4 concentrations were determined at IDEXX Reference Laboratories on Beckman AU clinical chemistry analyzers. SDMA was determined using a commercially available high-throughput immunoassay (IDEXX SDMA^®^ Test; IDEXX Laboratories Inc., One IDEXX Drive, Westbrook, Maine 04092, USA). CREA was determined by a colorimetric method, Jaffe’s reaction using picrate at alkaline pH (Beckman Coulter, Inc, Brea CA). Total T4 was determined by automated enzyme immunoassay (EIA) method to measure serum TT4 (DRI T4 assay; Microgenics, Freemont, CA, USA) [20]. Reference intervals for SDMA (0–14 μg/dL), CREA (0.9–2.3 μg/dL) and TT4 (0.8–4.7 μg/dL) in adult cats were previously established according to Clinical Laboratory and Standards Institute (CLSI) guidelines [21].

### Statistical analysis

Associations between TT4 and renal biomarkers were performed using a Spearman Rank correlation test in the hyperthyroid treated population. Descriptive statistical analysis was performed to document sequential changes in the renal biomarkers, CREA and SDMA, using medians and interquartile ranges at each sample time period for cats defined as the hyperthyroid treated population. Visual inspection of quantile-quantile plots indicated a non-normal distribution for CREA, SDMA and bodyweight thus the two-sided Mann Whitney U test was used to compare the distributions between the time periods among the hyperthyroid treated population and the age-matched control population. Since all time periods within the hyperthyroid treated group are paired, the two-sided Wilcoxon Signed-rank test was used along with the Holm-Bonferroni method for p-value adjustment to account familywise error rate in multiple testing [22]. To ensure that weight data were missing at random, the two-sample Kolmogorov-Smirnov test was used to compare the distributions of CREA, SDMA and age between the complete and incomplete weight data for the hyperthyroid treated cat population. Significance was set at an α of 0.05. All data analysis was performed with R version 3.3.1 [22]. Graphical analysis and data cleaning was performed using the *dplyr*, *ggsignif*, *ggpubr* and *ggplot2* R packages [23–26].

## Results

The study population is described in Fig 1. Definitions for each category are found in S1 Table. The baseline feline population of 453,126 cats had a total of 113,535 cats with complete SDMA, CREA, and TT4. A total of 37,431 cats made up the hyperthyroid cat group population with at least one visit where the cat had a TT4 value > 4.7 μg/dL. From the hyperthyroid cat group population, 4,680 cats met the criteria to be included in the hyperthyroid treated group and were used for analysis. The median age at pre-treatment visit was 14 years (range: 6–24 years). Domestic cats (Domestic Short Hair, Domestic Medium Hair, and Domestic Long Hair cats) made up 82% of the hyperthyroid treated group. The other four most common breeds were Siamese, American Short Hair, Maine Coon and Abyssinian, accounting for approximately 5% of the group. There were 10,358 sample visits; however, not all cats had a sample evaluated at each time-period and only 2,804 had visits that included complete body weight information (S2 Table).

### Correlation between SDMA, CREA and TT4

**Fig 2:**
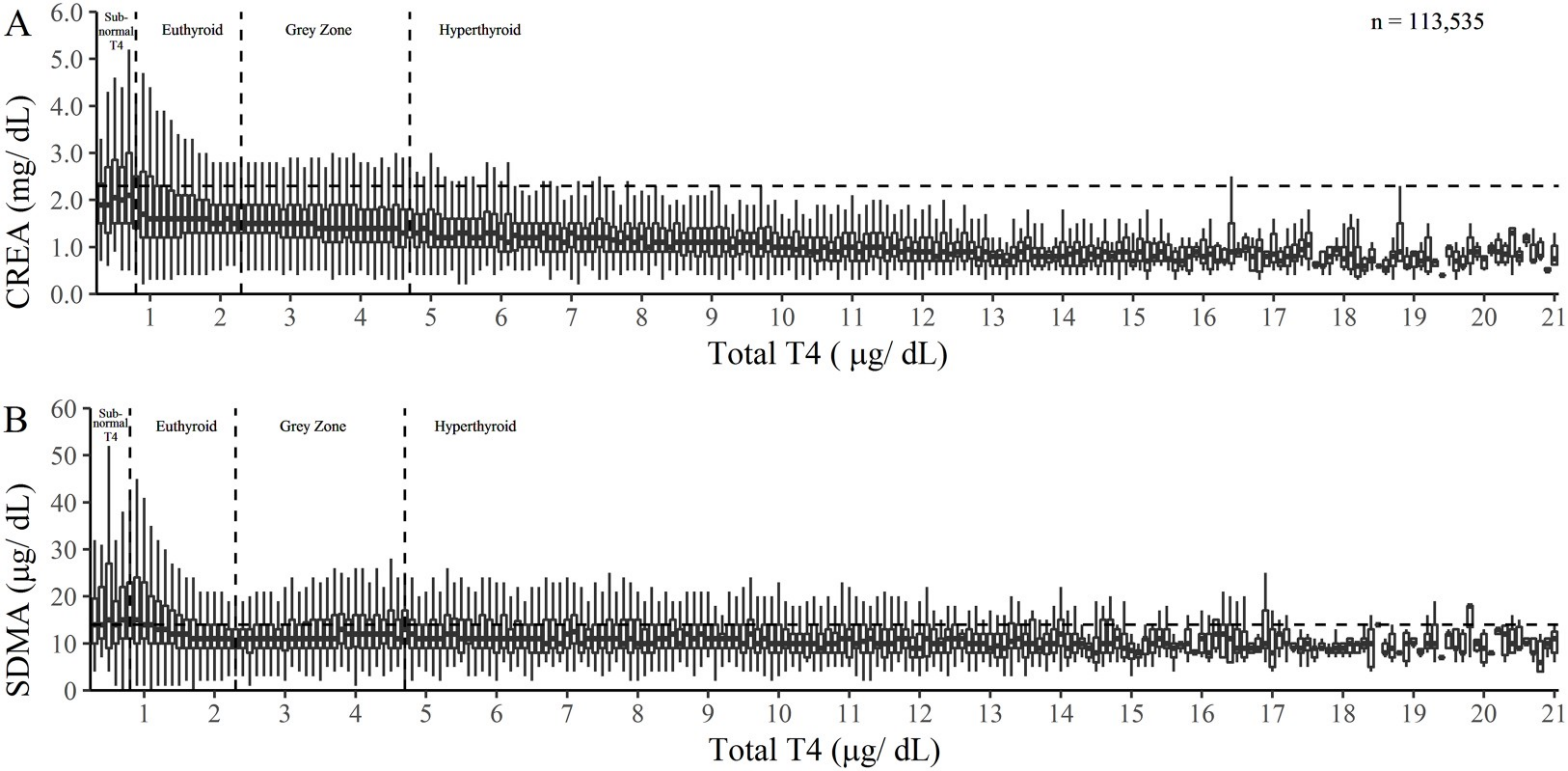
Relationship between TT4, SDMA and CRE concentrations in all cats with complete data (n = 113,535). 2A. Box plots illustrating the relationship between TT4 and CREA concentrations. 2B. Box plots illustrating the relationship between TT4 and SDMA concentrations.

SDMA and CREA were compared to TT4 concentrations for the entire study group (n = 113,535) and a series of boxplots were used to illustrate the relationship between TT4 and SDMA concentrations and similarly TT4 and CREA (Fig 2). When a Spearman rank correlation test was conducted for cats defined with hyperthyroidism (n = 4,680), a negative relationship was found between CREA and TT4 concentrations (Spearman’s ρ = -0.37, P < 0.001, S1 Figure). No relationship was found between SDMA and TT4 concentrations (Spearman’s ρ = 0.008, P = 0.426).

### Associations between hyperthyroidism and body weight

**Fig 3:**
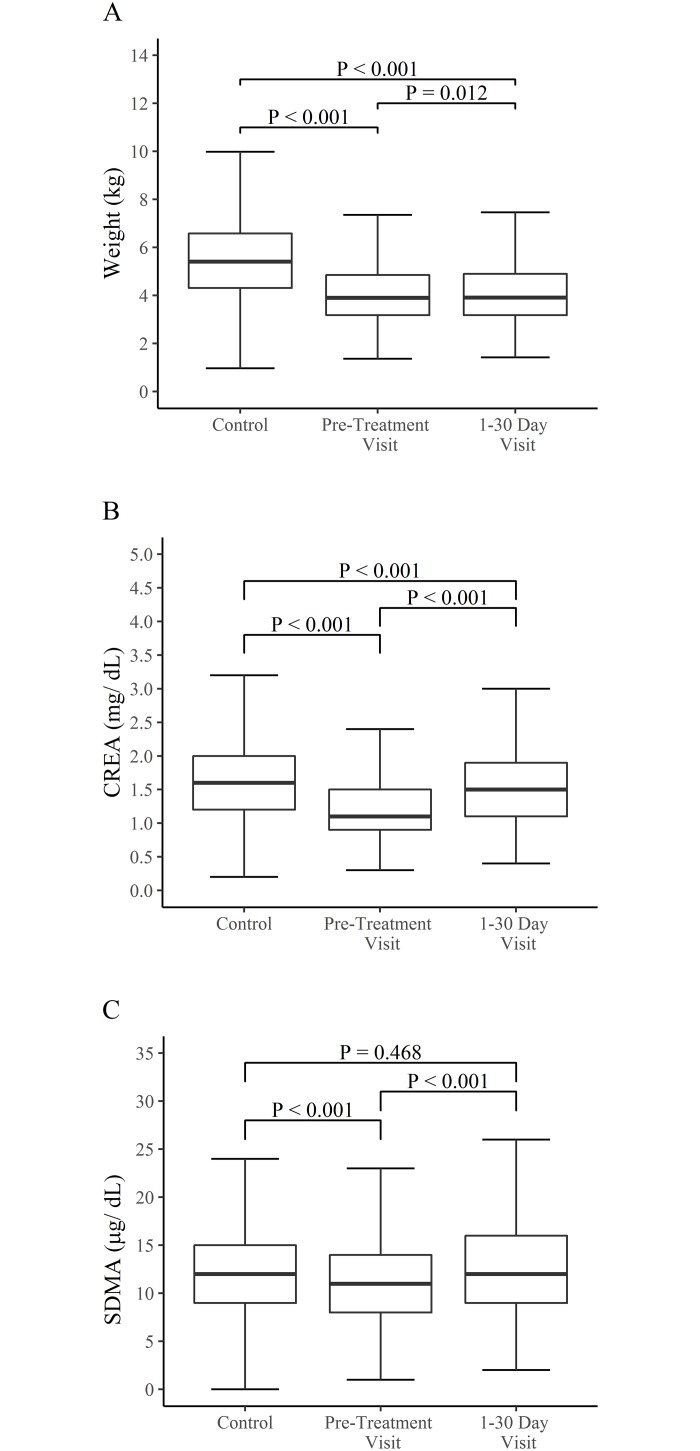
Comparison of the distributions of body weights, and SDMA and CREA concentrations between control cats (n = 12,810), pre-treatment visit hyperthyroid cats (n = 1,281), and first post-treatment visit hyperthyroid cats (n = 683). 3A. Changes in body weights. 3B. Changes in CREA concentrations. 3C. Changes in SDMA concentrations. Whiskers represent 1.5 times the interquartile range.

One thousand two hundred and eighty-one (1,281; 27.4%) of the hyperthyroid treated cats had body weights available for their pre-treatment visit. To assess whether the hyperthyroid treated cats with complete body weight information were representative of the entire hyperthyroid population, the difference in the distributions of CREA concentrations, SDMA concentrations, and age were tested between the two populations. There was no statistically significant difference between the distributions of SDMA, creatinine, and age between the cats that had their body weights recorded when compared to those cats that did not, (Kolmogorov-Smirnov test, P = 0.906, P = 0.822, and P = 0.205, respectively); supporting that this sub-group of hyperthyroid cats with recorded body weights was representative of the entire population of 4,680 hyperthyroid cats. When a comparison of the body weights of control euthyroid cats to hyperthyroid cats was performed using a 10 to 1 age-matched comparative analysis, cats with hyperthyroidism weighed less than control cats (Mann-Whitney U Test, P < 0.001, Fig 3A). Similarly, cats with hyperthyroidism had lower concentrations of both CREA and SDMA compared to control euthyroid cats (Mann-Whitney U Test, P < 0.001, Fig 3B and 3C).

CREA and SDMA concentrations are shown in Fig 3. Bodyweight, CREA, and SDMA concentrations increased significantly between the pre-treatment hyperthyroid state and the 1–30 day post-treatment visit (Wilcoxon Signed Rank Sum Test, P < 0.001, Fig 3, Table 1). CREA concentrations were significantly lower at the 1–30 day post-treatment visit compared to the control cats (Mann Whitney U Test, P < 0.001, Fig 3B, Table 1). SDMA concentrations at the 1–30 day post-treatment visit were not statistically different from controls (Mann Whitney U Test, P = 0.473, Fig 3C, Table 1).

**Table 1:** Descriptive statistics of SDMA, CREA, and bodyweight.

	N	Median	IQR	Min	Max	Mean	Standard Deviation	95% CI Around Mean	P-Value (91–120)	P-Value (Pre-Treat)
**SDMA (μg/dL)**										
**Control**	12810	12	12–12	1	100	14.49	10.24	14.31–14.67	0.114*	<0.001*
**Pre-Treatment**	1281	11	11–11	1	84	11.81	5.65	11.50–12.12	**<0.001**	**---**
**1–30 Days**	447	12	12–12	2	82	14.02	8.20	13.26–14.79	0.203	**<0.001**
**31–60 Days**	683	12	12–12	2	98	13.40	7.19	12.86–13.94	0.076	**<0.001**
**61–90 Days**	295	12	12–12	5	50	13.55	5.78	12.89–14.21	0.225	**<0.001**
**91–120 Days**	98	13	13–13	6	41	14.67	6.66	13.33–16.00	---	**<0.001**
**CREA (mg/dL)**										
**Control**	12810	1.6	1.6–1.6	0.2	26.9	1.89	1.43	1.87–1.92	0.170*	<0.001*
**Pre-Treatment**	1281	1.1	1.1–1.1	0.3	9.4	1.24	0.59	1.20–1.27	**<0.001**	**---**
**1–30 Days**	447	1.5	1.5–1.5	0.4	8.3	1.61	0.84	1.54–1.69	**<0.001**	**<0.001**
**31–60 Days**	683	1.5	1.5–1.5	0.2	11.2	1.66	0.78	1.60–1.71	**<0.001**	**<0.001**
**61–90 Days**	295	1.6	1.6–1.6	0.4	6.0	1.72	0.81	1.63–1.81	**0.007**	**<0.001**
**91–120 Days**	98	1.8	1.8–1.8	0.7	5.4	1.99	0.91	1.82–2.17	---	**<0.001**
**Body Weight (kg)**										
**Control**	12810	5.4	5.4–5.4	0.5	22.7	5.59	1.85	5.56–5.62	<0.001*	<0.001*
**Pre-Treatment**	1281	3.9	3.9–3.9	0.5	11.3	4.10	1.29	4.03–4.17	**0.006**	**---**
**1–30 Days**	447	3.9	3.9–3.9	1.4	10.0	4.09	1.28	3.98–4.21	**0.009**	0.960
**31–60 Days**	683	4.0	4.0–4.0	1.4	8.9	4.18	1.26	4.09–4.28	**0.043**	0.101
**61–90 Days**	295	4.1	4.1–4.1	1.4	11.3	4.25	1.34	4.10–4.41	0.124	0.064
**91–120 Days**	98	4.2	4.2–4.2	1.8	7.5	4.45	1.34	4.38–4.72	---	**0.006**

*All comparisons to the control group are done using the Mann–Whitney U test. All other comparisons are made using Wilcoxon signed-rank test. All multiple comparison p-values where adjusted using the Holm-Bonferroni method [22].

**Fig 4:**
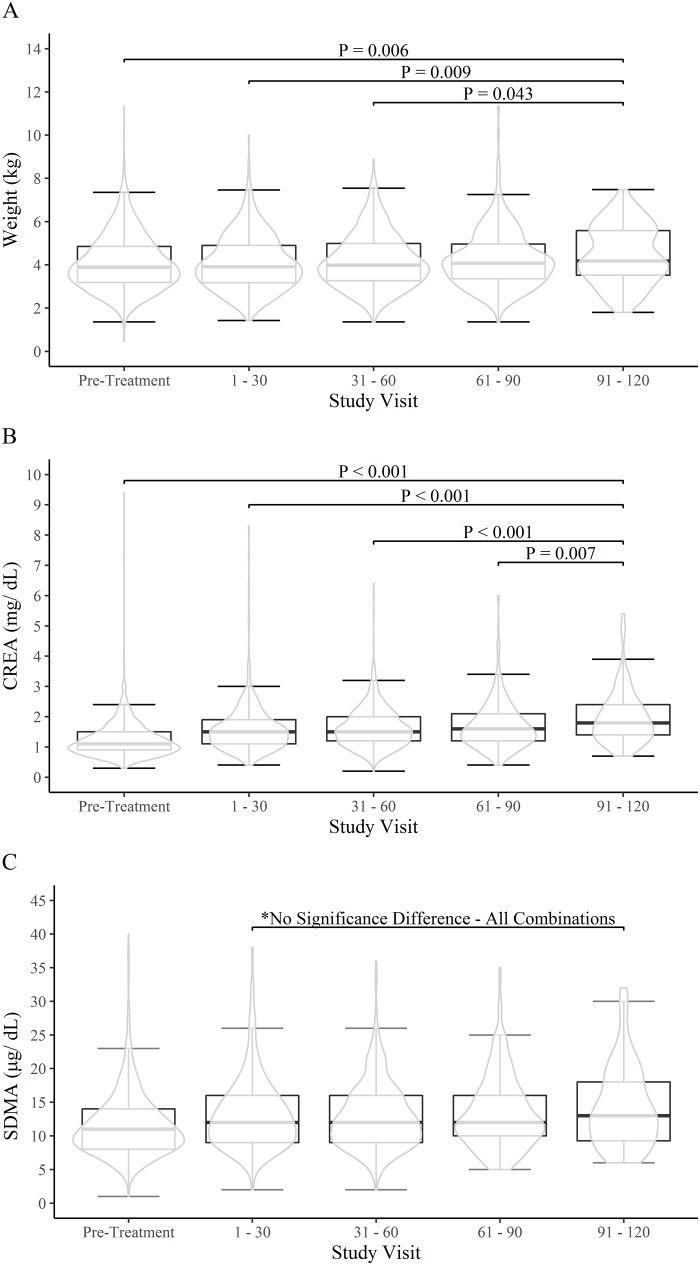
Comparison of the distributions of body weights, and SDMA and CREA concentrations between pre-treatment visit hyperthyroid cats (n = 1,281), 1–30 day post-treatment visit hyperthyroid cats (n = 447), 31–60 day post-treatment visit (n = 683), 61–90 day post-treatment visit (n = 295), and 91–120 day post-treatment visit (n = 98). 4A. Changes in body weights. 4B. Changes in CREA concentrations. 4C. Changes in SDMA concentrations. Whiskers represent 1.5 times the interquartile range.

The longer-term effect of treatment on body weight, CREA and SDMA concentrations are shown in Table 1. Hyperthyroid cats had significantly lower body weights pre-treatment, and at 1–30 days and 31–60 days versus 91–120 days (Wilcoxon Signed Rank Sum Test, all P values ≤ 0.043, Table 1, Fig 4A, S3 Table). Hyperthyroid cats had significantly lower CREA concentrations pre-treatment, and at 1–30 days, 31–60 days versus 91–120 days (Wilcoxon Signed Rank Sum Test, all P values ≤ 0.007, Table 1, S4 Table). Pre-treatment SDMA concentrations were significantly lower in comparison to all post-treatment time points (Wilcoxon Signed Rank Sum Test, all P values < 0.001, Table 1, Fig 4B, S5 Table). However, after the initial increase in SDMA concentrations at 1–30 days, there was no significant difference in SDMA concentrations throughout the remainder of the treatment periods (Table 1, Fig 4C, S6 Table). Comparisons across all combinations of time points can be found in S3–S5 Tables for bodyweight, CRE, and SDMA. Due to the small number of cats with visits at the 91–120 time point (n = 98), the distributions of CRE, SDMA, and age were compared for each time period in the group of cats with the 91–120 time and those without the 91–120 time point to ensure there was minimal survivorship bias. There was no statistically significant difference between the distributions of SDMA, creatinine, and age between the cats that had the 91–120 day time and those that did not have the 91–120 day (Kolmogorov-Smirnov test P > 0.05, all combinations of p-values (S6 Table).

## Discussion

Veterinarians rely heavily on kidney biomarkers such as CREA and blood urea nitrogen (BUN) to diagnose decreased renal function. These tests are nonspecific and can be affected by extra renal factors [13]. The purpose of this retrospective study was to describe the relationship between the renal biomarkers, SDMA and CREA, and TT4, in relation to body-weight changes in cats undergoing treatment of hyperthyroidism. Analysis of big data demonstrated that increased TT4 concentrations correlated with a decrease in CREA concentrations (Fig 2A). This finding is consistent with previous observations that kidney dysfunction may be masked by the hypermetabolic state of hyperthyroidism [27] and that reduced muscle mass results in reduced production of CREA [10]. By contrast, SDMA concentrations did not significantly decrease with increasing concentrations of TT4 (Fig 2B), suggesting that SDMA is more resistant to the effect of reduced muscle mass and increased GFR associated with increased TT4 concentrations than is CREA. A recent study on thyroidectomized hyperthyroid cats suggested a weak correlation between SDMA, bodyweight and TT4 levels [28]. In contrast, when pre-treatment cats were compared to control cats in the present study population, only pre-treatment cats had a significantly lower SDMA, and at no other time point was the SDMA concentration lower than the control population concentrations. This finding implies that if increased protein turnover resulting in increased SDMA production is occurring, it may be a relatively small effect.

It has been well established that the hyperthyroid state in cats causes an upregulation of GFR or hyperfiltration [5, 8, 10]. Several previous studies would suggest that as treatment is initiated, regardless of type, this upregulation of GFR is corrected around 30 days [5,10]. In this study SDMA did increase significantly between pre-treatment and 1–30 days in the treated population. This likely represents the correction of hyperfiltration and correlates with an expected normalization in GFR after treatment. The lack of significant difference between the SDMA concentrations at 1–30 days and the rest of the time periods likely reflects how minimal the effects of extrarenal factors may be on SDMA, especially those of such as muscle wasting and subsequent weight gain during treatment of the hyperthyroid state. A 1.5 kg weight difference was demonstrated between euthyroid controls and pre-treatment visit hyperthyroid cats (Fig 3A), and an age-matched comparison supports that this subgroup of hyperthyroid cats with recorded body weights was representative of the entire population of 4,680 hyperthyroid cats. On average, this represented a 27% reduction in body mass in the hyperthyroid population versus their age-matched counterparts. It is likely that a portion of this weight loss is due to reduced muscle mass, as thyrotoxicosis causes loss of muscle mass in the feline patient [29–31] and is a hallmark of hyperthyroidism, affecting over 75% of patients [9]. As muscle mass scores were not available in this retrospective study, changes in body weight were interpreted to be a proxy for the loss of muscle mass. Thus, in the study population a lower baseline weight in the hyperthyroid treated group served as a basis for evaluation of CREA and SDMA and the effect of reduced in muscle mass during treatment for hyperthyroidism.

A significant increase in body weights were seen subsequent to treatment, demonstrating that the study population appears to align with the known changes in muscle mass during therapy for hyperthyroidism [9]. Cat body weights increased an average of 0.4 kg by four months post hyperthyroid treatment (Table 1). This increase in weight may demonstrate recovery from a catabolic state and theoretically represents the potential change in muscle mass as these cats improve. Both CREA and SDMA concentrations increased in the initial 1–30 day time period, however only CREA concentrations continued to increase for months after GFR should have stabilized. This supports the conclusion that changes to muscle mass continued to affect CREA even 3 months post initiation of therapy for hyperthyroidism. Peterson et al demonstrated that a small percentage (10%) of appropriately treated cats remained thin, but almost half remained muscle wasted, and even in cats with follow up exceeding 6 months, muscle wasting was still present [6]. CREA concentrations therefore may remain an inadequate measure of renal function beyond achievement of euthyroid status.

There are several design limitations that are relevant to the interpretation of these results. In this study, the retrospective patient big data set have incomplete results for some parameters, irregular timepoints, potential bias toward frequent testing of sick patients, especially those with co-morbidities such as renal calculi, AKI events, iatrogenic hypothyroidism and other diseases or insults known to impact kidney function in addition to CKD. An additional limitation in this study was that mean body condition scores and muscle condition scores, or body mass index, were not available for the cats and body weight was used as a proxy for lean body mass. There were only 98 cats that had a follow-up time point at the 91–120 day time period, thus survivorship bias could be a factor in some of the results seen. We attempted to mitigate survivorship bias by comparing the distributions of SDMA and CRE between those cats that did and did not have a visit at the 91–120 day time period. Although no statistically significant difference between the distributions was seen, other variables not accounted for could be a factor in survivorship within the study. Further limitations are that other renal biomarkers such as urine specific gravity were not incorporated and that a subset of cats with a confirmed diagnosis of CKD was not identified. The population under study only included those patients that were considered effectively treated for hyperthyroidism and our data do not give us any insight into those that were not completely treated or not treated at all. In addition, we converted the time post-treatment from a continuous variable into an ordinal variable with defined bins reflecting key clinical follow-up times. Though this might aid in the interpretability for clinical viewers, it decreases the power and precision of our estimates. Multivariate or simultaneous analysis of the biomarkers was not performed since it is beyond the scope of the present work. Despite these limitations, big data allowed laboratory results from a large feline population to be collected and evaluation of multiple of data points provided by each individual patient and visit to be analyzed to best define statistically significant trends leading to further medical insights.

To better understand the relationship of these biomarkers, further work is needed to define a subgroup of hyperthyroid cats diagnosed with renal dysfunction with the goal of establishing the relative reliability and strength of CREA and SDMA for confirmation, monitoring, and management of the hyperthyroid cat with renal dysfunction, including CKD. This information may facilitate earlier discovery of renal dysfunction and avoidance of over-treatment, which are important for patient quality of life and prognosis [32].

## Conclusion

The goal of this study was to present descriptive data to help support current hypotheses about renal biomarkers and hyperthyroidism. Our findings suggest that CREA concentrations significantly decreased with increasing concentrations of TT4, whereas SDMA did not. In cats treated for hyperthyroidism both CREA and SDMA concentrations increased in the immediate post-treatment period, indicating resolution of hyperfiltration and reduction in GFR. After this initial increase post-treatment, SDMA concentrations remained stable during the post-treatment period. CREA concentrations, however, continued to increase throughout the extended post-treatment period supporting the argument that extrarenal factors such as muscle mass influence CREA in this disease state. Further work is needed to define a subgroup of hyperthyroid cats with CKD and study how CREA and SDMA concentrations are affected by hyperthyroid treatment to better understand these relationships and clinical ramifications. This study supports that SDMA is less affected by extrarenal factors than CRE in hyperthyroid cats before and after treatment.

## Supporting information

S1 Table Definitions. (DOCX)

S2 Table Number of unique visits for each time-period. (DOCX)

S3 Table Comparison of distributions of bodyweight by visit. (DOCX)

S4 Table Comparison of distributions of CREA by visit. (DOCX)

S5 Table Comparison of distributions of SDMA by visit. (DOCX)

S6 Table Comparison of distributions of SDMA, CRE, and age by cats that had a 91–120 day time point to those that did not. (DOCX)

S1 Figure Relationship between TT4 and CRE concentrations in all cats defined as hyperthyroid (n = 4,680). (TIFF)

S1 Dataset SDMA and CREA results for control (euthyroid) cats. (CSV)

S2 Dataset SDMA, CREA, body weight and time bin for hyperthyroid cats. (CSV)

## References

[1] Wakeling J, Elliott J, Syme H. Evaluation of predictors for the diagnosis of hyperthyroidism in cats. J Vet Intern Med. 2011;25: 1057–1065. 10.1111/j.1939-1676.2011.00790.x 21985139

[2] Peterson M. Hyperthyroidism in cats: what’s causing this epidemic of thyroid disease and can we prevent it? J Feline Med Surg. 2012;14: 804–818. 10.1177/1098612X12464462 23087006 PMC11112171

[3] Riensche MR, Graves TK, Schaeffer DJ. An investigation of predictors of renal insufficiency following treatment of hyperthyroidism in cats. J Feline Med Surg. 2008;10: 160–166. 10.1016/j.jfms.2007.10.005 18086546 PMC10911216

[4] Becker TJ, Graves TK, Kruger JM, Braselton WE, Nachreiner RF. Effects of methimazole on renal function in cats with hyperthyroidism. J Am Anim Hosp Assoc. 2000;36: 215–223. 10.5326/15473317-36-3-215 10825092

[5] Boag AK, Neiger R, Slater L, Stevens KB, Haller M, Church DB. Changes in the glomerular filtration rate of 27 cats with hyperthyroidism after treatment with radioactive iodine. Vet Rec. 2007;161: 711–715. 10.1136/vr.161.21.711 18037692

[6] Milner RJ, Channell CD, Levy JK, Schaer M. Survival times for cats with hyperthyroidism treated with iodine 131, methimazole, or both: 167 cases (1996–2003). Journal of the American Veterinary Medical Association. 2006;228: 559–563. 10.2460/javma.228.4.559 16478432

[7] Adams WH, Daniel GB, Legendre AM, Gompf RE, Grove CA. Changes in renal function in cats following treatment of hyperthyroidism using 131I. Vet Radiol Ultrasound. 1997;38: 231–238. 10.1111/j.1740-8261.1997.tb00846.x 9238796

[8] Graves TK, Olivier NB, Nachreiner RF, Kruger JM, Walshaw R, Stickle RL. Changes in renal function associated with treatment of hyperthyroidism in cats. Am J Vet Res. 1994;55: 1745–1749. 7887521

[9] Peterson ME, Castellano CA, Rishniw M. Evaluation of Body Weight, Body Condition, and Muscle Condition in Cats with Hyperthyroidism. J Vet Intern Med. 2016;30: 1780–1789. 10.1111/jvim.14591 27667652 PMC5115195

[10] Vaske HH, Schermerhorn T, Grauer GF. Effects of feline hyperthyroidism on kidney function: a review. J Feline Med Surg. 2016;18: 55–59. 10.1177/1098612X15575385 25749888 PMC11149009

[11] Baxmann AC, Ahmed MS, Marques NC, Menon VB, Pereira AB, Kirsztajn GM, et al Influence of muscle mass and physical activity on serum and urinary creatinine and serum cystatin C. Clin J Am Soc Nephrol. 2008;3: 348–354. 10.2215/CJN.02870707 18235143 PMC2390952

[12] Wakeling J, Moore K, Elliott J, Syme H. Diagnosis of hyperthyroidism in cats with mild chronic kidney disease. Journal of Small Animal Practice. 2008;49: 287–294. 10.1111/j.1748-5827.2008.00544.x 18422499

[13] Hall JA, Yerramilli M, Obare E, Yerramilli M, Jewell DE. Comparison of serum concentrations of symmetric dimethylarginine and creatinine as kidney function biomarkers in cats with chronic kidney disease. J Vet Intern Med. 2014;28: 1676–1683. 10.1111/jvim.12445 25231385 PMC4895610

[14] Nabity MB, Lees GE, Boggess MM, Yerramilli M, Obare E, Yerramilli M, et al Symmetric Dimethylarginine Assay Validation, Stability, and Evaluation as a Marker for the Early Detection of Chronic Kidney Disease in Dogs. J Vet Intern Med. 2015;29: 1036–1044. 10.1111/jvim.12835 26079532 PMC4895368

[15] Hall JA, Yerramilli M, Obare E, Yerramilli M, Almes K, Jewell DE. Serum Concentrations of Symmetric Dimethylarginine and Creatinine in Dogs with Naturally Occurring Chronic Kidney Disease. J Vet Intern Med. 2016;30: 794–802. 10.1111/jvim.13942 27103204 PMC4913574

[16] Schwedhelm E, Böger RH. The role of asymmetric and symmetric dimethylarginines in renal disease. Nat Rev Nephrol. 2011;7: 275–285. 10.1038/nrneph.2011.31 21445101

[17] Kielstein JT, Böger RH, Bode-Böger SM, Frölich JC, Haller H, Ritz E, et al Marked increase of asymmetric dimethylarginine in patients with incipient primary chronic renal disease. J Am Soc Nephrol. 2002;13: 170–176. 11752034 10.1681/ASN.V131170

[18] Hall JA, Yerramilli M, Obare E, Yerramilli M, Melendez LD, Jewell DE. Relationship between lean body mass and serum renal biomarkers in healthy dogs. J Vet Intern Med. 2015;29: 808–814. 10.1111/jvim.12607 25913398 PMC4895404

[19] Billings. Nonlinear System Identification: NARMAX Methods in the Time, Frequency, and Spatio-Temporal Domains. In: Wiley.com [Internet]. [cited 15 Jan 2019]. https://www.wiley.com/en-us/Nonlinear+System+Identification%3A+NARMAX+Methods+in+the+Time%2C+Frequency%2C+and+Spatio+Temporal+Domains-p-9781119943594

[20] Williams TL, Archer J. Validation of an automated enzyme immunoassay for the measurement of serum total thyroxine in cats. Vet Clin Pathol. 2016;45: 148–153. 10.1111/vcp.12324 26840919

[21] NCLSI. Clinical Laboratory Standards Institue Manual. 2008.

[22] R Development Core Team. R: A Language and Environment for Statistical Computing [Internet]. Vienna, Austria: R Foundation for Statistical Computing; 2009. http://www.R-project.org

[23] Ahlmann-Eltze C. ggsignif: Significance Brackets for “ggplot2” [Internet]. 2017. https://CRAN.R-project.org/package=ggsignif

[24] Wickham H, Chang W, Henry L, Pedersen TL, Takahashi K, Wilke C, et al. ggplot2: Create Elegant Data Visualisations Using the Grammar of Graphics [Internet]. 2018. https://CRAN.R-project.org/package=ggplot2

[25] Wickham H, François R, Henry L, Müller K, RStudio. dplyr: A Grammar of Data Manipulation [Internet]. 2018. https://CRAN.R-project.org/package=dplyr

[26] Kassambara A. ggpubr: “ggplot2” Based Publication Ready Plots [Internet]. 2019. https://CRAN.R-project.org/package=ggpubr

[27] van Hoek I, Lefebvre HP, Kooistra HS, Croubels S, Binst D, Peremans K, et al Plasma clearance of exogenous creatinine, exo-iohexol, and endo-iohexol in hyperthyroid cats before and after treatment with radioiodine. J Vet Intern Med. 2008;22: 879–885. 10.1111/j.1939-1676.2008.0110.x 18498324

[28] Covey HL, Chang Y, Elliott J, Syme HM. Changes in thyroid and renal function after bilateral thyroidectomy in cats. Journal of Veterinary Internal Medicine. 2019;0 10.1111/jvim.15450 30758070 PMC6430951

[29] Thoday KL, Mooney CT. Historical, clinical and laboratory features of 126 hyperthyroid cats. Vet Rec. 1992;131: 257–264. 10.1136/vr.131.12.257 1413411

[30] Peterson ME, Kintzer PP, Cavanagh PG, Fox PR, Ferguson DC, Johnson GF, et al Feline hyperthyroidism: pretreatment clinical and laboratory evaluation of 131 cases. J Am Vet Med Assoc. 1983;183: 103–110. 6874510

[31] Holzworth J, Theran P, Carpenter JL, Harpster NK, Todoroff RJ. Hyperthyroidism in the cat: ten cases. J Am Vet Med Assoc. 1980;176: 345–353. 7358553

[32] Williams TL, Elliott J, Syme HM. Association of iatrogenic hypothyroidism with azotemia and reduced survival time in cats treated for hyperthyroidism. J Vet Intern Med. 2010;24: 1086–1092. 10.1111/j.1939-1676.2010.0566.x 20695989

