# Supplementary material for: A retrospective evaluation of the relationship between symmetric dimethylarginine, creatinine and body weight in hyperthyroid cats
Source: PLoS One. 2020 Jan 28;15(1):e0227964. doi: 10.1371/journal.pone.0227964 (PMC6986741; doi:10.1371/journal.pone.0227964)
Supplement: S1 Table — (DOCX) [file pone.0227964.s001.docx]

| **Term** | **Definition** |
| --- | --- |
| **Big Data** | Large data sets with low information density, that use computer based statistical methods to reveal relationships and dependencies within populations, and perform predictions of outcomes and behaviors. |
| **Sub-normal cat** | Cat, 6 to 25 years of age, with a TT4 concentration < 0.8 µg/dL |
| **Euthyroid Control cat** | Cat, 6 to 25 years of age, with a TT4 concentration between 0.8 and 2.2 µg/dL |
| **‘Grey zone’ cat** | Cat, 6 to 25 years of age, a TT4 concentration between 2.3 and 4.7 µg/dL |
| **Hyperthyroid cat** | Cat, 6 to 25 years of age, a TT4 concentration > 4.7 µg/dL |
| **Hyperthyroid treated cat** | Hyperthyroid cat where TT4 concentrations in subsequent samples have decreased by a minimum of 2.5 µg/dL and are ≤ 4.7 µg/dL. |
| **Pre-treatment visit** | The date at which the cat’s sample had a TT4 concentration > 4.7 µg/dL |
| **Post-treatment visit(s)** | The date(s) at which the cat’s sample had a TT4 concentration ≤ 4.7 µg/dL with a minimum decrease of 2.5 µg/dL from pre-treatment levels. |
