# Supplementary material for: A retrospective evaluation of the relationship between symmetric dimethylarginine, creatinine and body weight in hyperthyroid cats
Source: PLoS One. 2020 Jan 28;15(1):e0227964. doi: 10.1371/journal.pone.0227964 (PMC6986741; doi:10.1371/journal.pone.0227964)
Supplement: S2 Table — (DOCX) [file pone.0227964.s002.docx]

| Time Period | Frequency | Frequency  (complete BW information) |
| --- | --- | --- |
| Pre-treatment | 4,680 | 1,281 |
| 1-30 | 1,695 | 447 |
| 31-60 | 2,526 | 683 |
| 61-90 | 1,080 | 295 |
| 91-120 | 377 | 98 |
| Total | 10,358 | 2,804 |
