# Supplementary material for: A retrospective evaluation of the relationship between symmetric dimethylarginine, creatinine and body weight in hyperthyroid cats
Source: PLoS One. 2020 Jan 28;15(1):e0227964. doi: 10.1371/journal.pone.0227964 (PMC6986741; doi:10.1371/journal.pone.0227964)
Supplement: S3 Table — (DOCX) [file pone.0227964.s003.docx]

| **Bodyweight (kg)** | **Pre-treatment** | **Post-treatment (days)** | | | |
| --- | --- | --- | --- | --- | --- |
|  |  | **1-30** | **31-60** | **61-90** | **91-120** |
| **Control*** | **<0.001*** | **<0.001*** | **<0.001*** | **<0.001*** | **<0.001*** |
| **Pre-treatment** | --- | 0.960 | 0.101 | 0.064 | **0.006** |
| **1-30** |  | --- | 0.419 | 0.220 | **0.009** |
| **31-60** |  |  | --- | 0.546 | **0.043** |
| **61-90** |  |  |  | --- | 0.124 |
| **91-120** |  |  |  |  | --- |

*All comparisons to the control group are done using the Mann–Whitney U test.

All other comparisons are made using Wilcoxon signed-rank test.
