# Supplementary material for: A retrospective evaluation of the relationship between symmetric dimethylarginine, creatinine and body weight in hyperthyroid cats
Source: PLoS One. 2020 Jan 28;15(1):e0227964. doi: 10.1371/journal.pone.0227964 (PMC6986741; doi:10.1371/journal.pone.0227964)
Supplement: S6 Table — (DOCX) [file pone.0227964.s006.docx]

| **Variable** | **Time-Period** | **D** | **P-Value*** |
| --- | --- | --- | --- |
| **SDMA** |  |  |  |
| **Pre-Treatment** | | 0.043 | 0.066 |
| **1-30** | | 0.067 | 0.095 |
| **31-60** | | 0.035 | 0.482 |
| **91-120** | | 0.016 | 0.863 |
| **CRE** |  |  |  |
| **Pre-Treatment** | | 0.016 | 0.605 |
| **1-30** | | 0.037 | 0.388 |
| **31-60** | | 0.011 | 0.878 |
| **91-120** | | 0.005 | 0.987 |
| **Age** |  |  |  |
| **Pre-Treatment** | | 0.005 | 0.947 |
| **1-30** | | 0.014 | 0.876 |
| **31-60** | | 0.003 | 0.989 |
| **91-120** | | 0.049 | 0.302 |

*Kolmogorov-Smirnov test.
