# Supplementary figures and images for: A retrospective evaluation of the relationship between symmetric dimethylarginine, creatinine and body weight in hyperthyroid cats
Source: PLoS One. 2020 Jan 28;15(1):e0227964. doi: 10.1371/journal.pone.0227964 (PMC6986741; doi:10.1371/journal.pone.0227964)

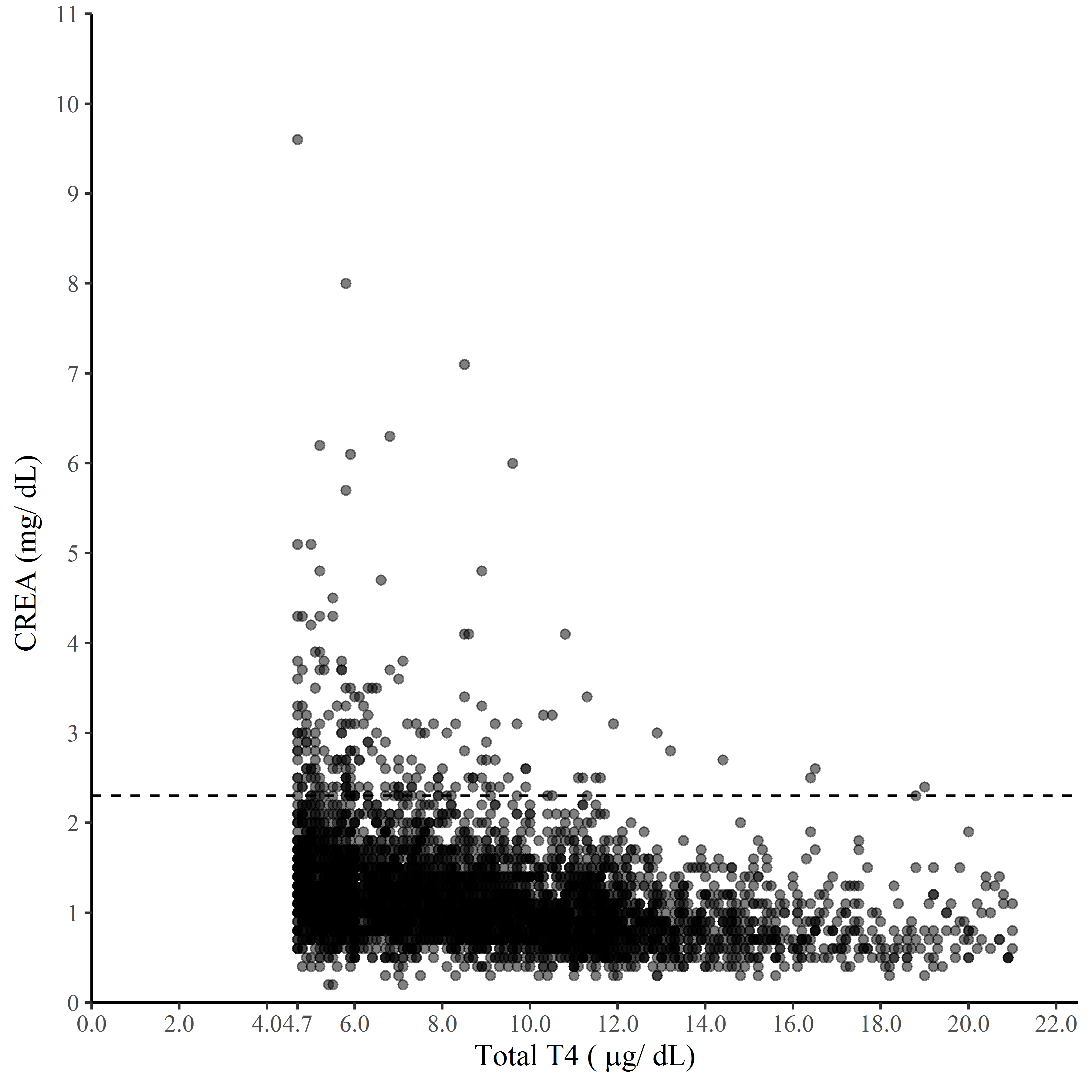

Supplement: S1 Figure — (TIFF) [file pone.0227964.s007.tiff]
